# Prevention of Chemotherapy-Induced Peripheral Neuropathy by Inhibiting C-X-C Motif Chemokine Receptor 2

**DOI:** 10.3390/ijms24031855

**Published:** 2023-01-17

**Authors:** Hee Seong Cho, Young In Choi, Seon Uk Park, Yi Seul Han, Jean Kwon, Sung Jun Jung

**Affiliations:** 1 Department of Biomedical Science, Graduate School of Biomedical Science and Engineering, Hanyang University, Seongdong-gu, Seoul 04763, Republic of Korea; 2 Department of Biological Sciences, Columbia University, New York, NY 10027, USA; 3 Department of Physiology, College of Medicine, Hanyang University, Seongdong-gu, Seoul 04763, Republic of Korea

**Keywords:** vincristine, oxaliplatin, CXCR2, CIPN, reparixin, prevention

## Abstract

Chemotherapy-induced peripheral neuropathy (CIPN) is a major drawback in the use of chemotherapeutic agents for patients with cancer. Although studies have investigated a broad number of molecules that might be related to CIPN, the differences in the chemokine pathways between various chemotherapeutic agents, such as vincristine and oxaliplatin, which are some of the most widely used treatments, have not been fully elucidated. We confirmed that the administration (intraperitoneal injections for seven days) of vincristine (0.1 mg/kg) and oxaliplatin (3 mg/kg) induced pain by using the von Frey behavioral test. Subsequent applications with vincristine and oxaliplatin led to mechanical allodynia that lasted more than one week from the fifth day. After the induction of mechanical allodynia, the mRNA expression of CXCR2, CXCL1, CXCL3, and CXCL5 was examined in the dorsal root ganglia (DRG) and spinal cord of the CIPN models. As a result, the mRNA expression of CXCR2 robustly increased in the lumbar spinal cord in the oxaliplatin-treated mice. Next, to evaluate the involvement of CXCR2 in CIPN, reparixin, a CXCR1/2 inhibitor, was administered intrathecally or intraperitoneally with vincristine or oxaliplatin and was further verified by treatment with ruxolitinib, which inhibits Janus kinase 2 downstream of the CXCR1/2 pathway. Reparixin and ruxolitinib blocked oxaliplatin-induced allodynia but not vincristine-induced allodynia, which suggests that CXCR2-related pathways are associated with the development of oxaliplatin-induced neuropathy. Together with the above results, this suggests that the prevention of oxaliplatin-induced neuropathy by CXCR2 inhibition can lead to successful chemotherapy, and it is important to provide appropriate countermeasures against CIPN development for each specific chemotherapeutic agent.

## 1. Introduction

Chemotherapy-induced peripheral neuropathy (CIPN) frequently occurs in patients treated with anticancer drugs, including platinum derivatives, taxanes, vinca alkaloids, and thalidomide [1]. Approximately more than 50% of patients under chemotherapy experience CIPN, but the ratio of incidence differs depending on the type and dose of anticancer drug [2]. CIPN manifests as paresthesia, dysesthesia, and numbness in the hands and feet through chemotherapeutic damage to long peripheral axons [3]. These symptoms are characterized by the development of severe peripheral pain during chemotherapy treatment or long-lasting and persistent pain after the end of chemotherapy [4]. Although treatment for chronic persistent pain after chemotherapy is also necessary, the occurrence of CIPN in some patients undergoing chemotherapy may lead to discontinuation of chemotherapy; thus, the prevention of CIPN occurrence during chemotherapy is an important issue [5]. However, the molecular mechanism behind each drug remains elusive, despite continuous efforts to overcome CIPN induced by various chemotherapeutic agents [6].

Numerous investigations have identified a variety of molecules and pathways that may contribute to the development of CIPN [7], and both prevention and treatment have been considered as two possible suggestions for CIPN management. At present, reductions in the cumulative and individual dose intensities are considered to effectively prevent CIPN, but no drug can claim to prevent CIPN [8] due to the lack of mechanism studies on CIPN development. Currently, general therapy, including the administration of lidocaine, opioids, and SSRNI, is applied for pain management [1]. Therefore, a full understanding of the involved pathway is necessary to suggest possible methods for the prevention or treatment of CIPN.

Oxaliplatin, known to cause frequent CIPN as a platinum-based chemotherapeutic agent, acts by binding platinum to neuronal DNA [9] and it has been used as a chemotherapeutic agent to treat colorectal and esophageal cancer [10,11,12,13]. Most patients treated with oxaliplatin (94%) experience cold allodynia and 87% of them develop acute symptoms, while the use of oxaliplatin carries a 50% chance of potential chronic CIPN development after four weeks of treatment [14]. Among the widely used anticancer drugs, vincristine, a vinca alkaloid family chemotherapeutic agent, acts by interfering with the formation of mitotic spindles and the assembly of microtubules, which causes damage to cell division and leads to apoptosis [5]. Regardless of its effectiveness as a chemotherapeutic agent to treat leukemia, non-Hodgkin’s lymphoma, Hodgkin’s disease, and some solid tumors [15,16,17,18], 30–40% of patients experience the development of CIPN [5,19], which could last for more than four years, and such chronic CIPN is difficult to cure [20].

The peripheral neuropathy induced by these two chemotherapeutic agents is associated with chemokine pathways [6]. Various chemokines that are released from macrophages or glial cells are reported to be key molecules in vincristine- or oxaliplatin-induced neuropathy, as well as in neuropathic pain [21,22,23]. Previous studies suggested that the administration of vincristine resulted in NFκB-dependent CXCL1/CXCR2 signaling [24] and CCL2/CCR2 signaling-dependent monocyte infiltration into the sciatic nerve [25] and that the administration of oxaliplatin resulted in the activation of the T-cell (NFAT)-CCR2 pathway [26], and microglia–astrocyte-induced the IL8/CXCR2 pathway [27]. In addition, the upregulation of CXCL9, CXCL10, IL6, IL18, IL1β, and TNF-α and the infiltration of the CX3CR1+ macrophages in the sciatic nerve are associated with vincristine-induced neuropathy [28,29,30], while the administration of oxaliplatin can decrease CCL4 in DRG, and increase CD45+CD4+ and CD45+CD8 in the blood and IL4 in the spleen [31]. Although both anticancer drugs induce similar peripheral neuropathy, it is not known whether the chemokines involved are similar or different.

In this study, we investigated the difference in the relevance of chemokines in the development of peripheral neuropathy caused by the administration of vincristine and oxaliplatin, and we propose an approach that can prevent the occurrence of CIPN based on chemokine-related mechanisms.

## 2. Results

### 2.1. Vincristine Induces Peripheral Inflammation along with CIPN

**Figure 1:**
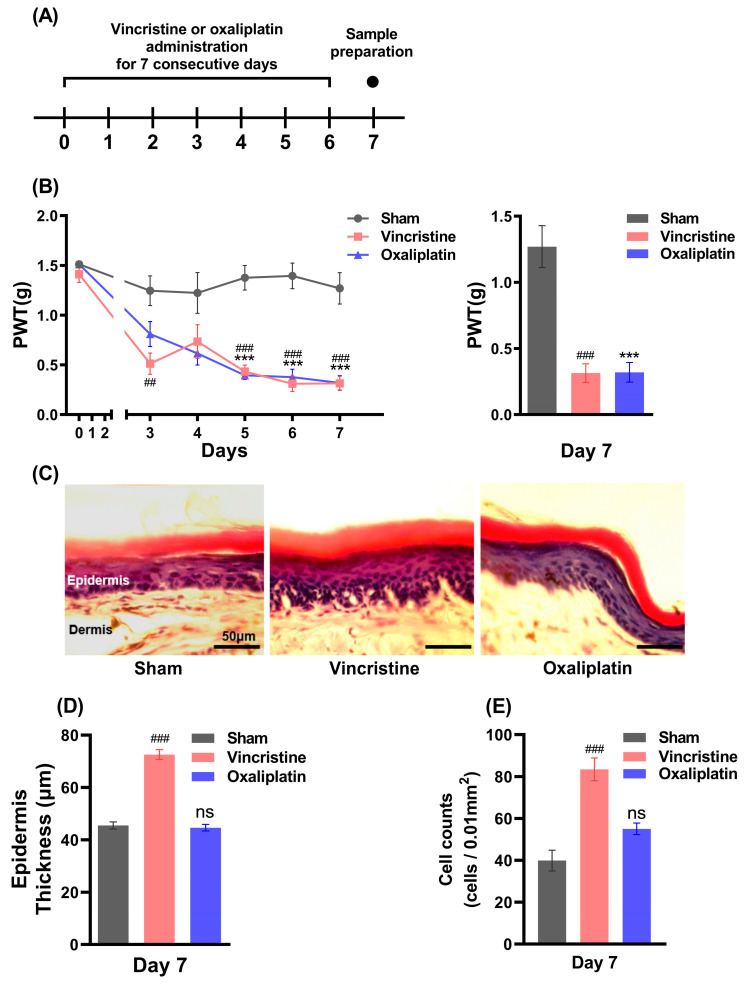
(**A**) Injection scheme to induce CIPN. (**B**) PWT measured for mechanical allodynia test using von Frey filament and PWT on the 7th day of the experiment (n = 5). (**C**) Histology of hind paw skin of a mouse on 7th day; scale bar represents 50 μm in length (n = 4). (**D**) Epidermal thickness length measured. Vincristine- and oxaliplatin-treated mice were compared to the sham mice. (**E**) Number of cells counted per 0.01 mm^2^ in the epidermis and dermis layer (n = 9). Results are presented as the mean ± SEM (## *p* < 0.01, ### *p* < 0.001, *** *p* < 0.001, ANOVA, followed by Bonferroni post hoc test).

Vincristine, oxaliplatin, and normal saline were each injected intraperitoneally for 7 days (Figure 1A), and after measuring the baseline of the paw withdrawal threshold (PWT) on day 0, the PWT was measured 24 h after each drug injection. The PWT was reduced in the vincristine- and oxaliplatin-treated mice compared to the sham mice from the 5th day, and the PWT measured on the 7th day (statistically) significantly decreased, indicating that mechanical allodynia occurred: 1.270 ± 0.158 g in the sham mice, 0.314 ± 0.071 g in the vincristine-treated mice, and 0.320 ± 0.074 g in the oxaliplatin-treated mice (Figure 1B, n = 5). After the full development of CIPN, the epidermal thickness of the mouse hind paws on the 7th day was measured (Figure 1C, n = 4), as previously described by Cheng et al. (2020) [32]. The epidermal thickness of the hind paws in the vincristine-treated mice was 72.60 ± 1.88 μm, which was thicker than that of both the sham mice and oxaliplatin-treated mice, with 45.48 ± 1.35 μm and 44.65 ± 1.26 μm, respectively (Figure 1D). The number of infiltrated cells was counted, as previously described by Boman et al. (1996) [33]. In the vincristine-treated mice, 83.44 ± 5.40 cells/0.01 mm^2^ of infiltrated cells was observed, which was significantly more than the sham mice and oxaliplatin-treated mice: 39.89 ± 4.97 cells/0.01 mm^2^ and 55.00 ± 2.75 cells/0.01 mm^2^, respectively (Figure 1E, n = 9). These results indicate the difference in the peripheral phenotype between the vincristine- and oxaliplatin-treated mice; nonetheless, the development of CIPN was induced by both chemotherapeutic agents. Although CIPN develops after vincristine and oxaliplatin administration, the results showing the different epidermal changes suggest that the pain mechanisms involved may be different.

### 2.2. Increases in CXCR2 mRNA in the Lumbar Spinal Cord in the Early Stage of Oxaliplatin-Induced Neuropathy

**Figure 2:**
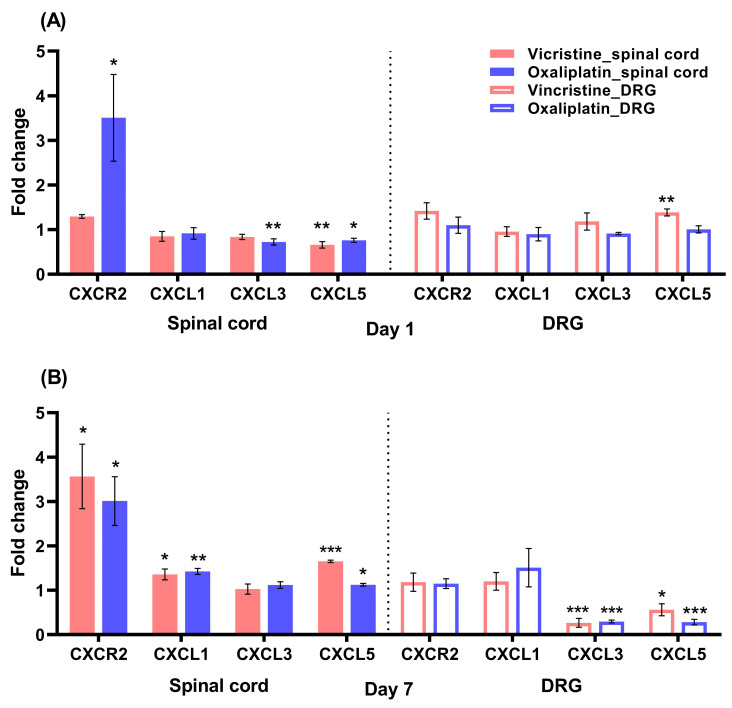
(**A**) Real-time quantitative PCR results showing mRNA expression levels of CXCR2, CXCL1, CXCL3, and CXCL5 in the lumbar spinal cord and DRG after the 1st day from the first injection of either vincristine or oxaliplatin (n = 4). (**B**) mRNA expression levels of targets in the lumbar spinal cord and DRG after the 7th day from the first injection of either vincristine or oxaliplatin (n = 4). All the expression levels were normalized to the amount of GAPDH and were compared to the sham group. Results are presented as the mean relative fold change ± SEM (* *p* ≤ 0.05, ** *p* ≤ 0.01, *** *p* ≤ 0.001, ANOVA followed by Bonferroni post hoc test).

Next, we investigated which chemokines in the spinal cord and DRG were involved in CIPN. The mRNA expression of chemokines was evaluated in the spinal cord and DRG of each CIPN model (n = 4). The fold change of CXCR2 expression in the lumbar spinal cord increased after the 1st day of oxaliplatin administration by 3.506 ± 0.971-fold, while the mRNA expression of CXCR2 did not alter with a 1.295 ± 0.039-fold change after vincristine administration when compared to the sham group; CXCL3 expression decreased after oxaliplatin administration by 0.723 ± 0.069-fold, and CXCL5 expression decreased after the administration of both vincristine and oxaliplatin by 0.659 ± 0.072-fold and 0.759 ± 0.608-fold, respectively (Figure 2A). On the 7th day, the CXCR2 mRNA expression in the lumbar spinal cord increased after both vincristine and oxaliplatin administration by 3.564 ± 0.724-fold and 3.010 ± 0.549-fold, respectively (Figure 2B), which is in line with other research, proving the increase in CXCR2 in both vincristine- and oxaliplatin-induced neuropathy [24,27]. Additionally, CXCL1 and CXCL5 expression increased after both vincristine and oxaliplatin administration (CXCL1: 1.356 ± 0.123-fold and 1.426 ± 0.068-fold, respectively; CXCL5: 1.651 ± 0.030-fold and 1.125 ± 0.031-fold, respectively). As regards the change in the chemokine mRNA of DRG, no significant changes in both the vincristine- and oxaliplatin-treated mice were observed on the 1st day, except for an increase in CXCL5 expression after vincristine administration by 1.386 ± 0.078-fold, but vincristine and oxaliplatin administration both decreased the mRNA expression of CXCL3 and CXCL5 on the 7th day (CXCL3: 0.266 ± 0.098-fold and 0.295 ± 0.031-fold, respectively; CXCL5: 0.562 ± 0.135-fold and 0.282 ± 0.063-fold, respectively). These results suggest that oxaliplatin administration increased CXCR2 from the beginning of the pain and maintained it consistently, while vincristine administration only involved CXCR2 in the late phase of the pain (7th day) after the CIPN had already developed.

### 2.3. Inhibition of CXCR2 with Reparixin Prevents Oxaliplatin-Induced CIPN

**Figure 3:**
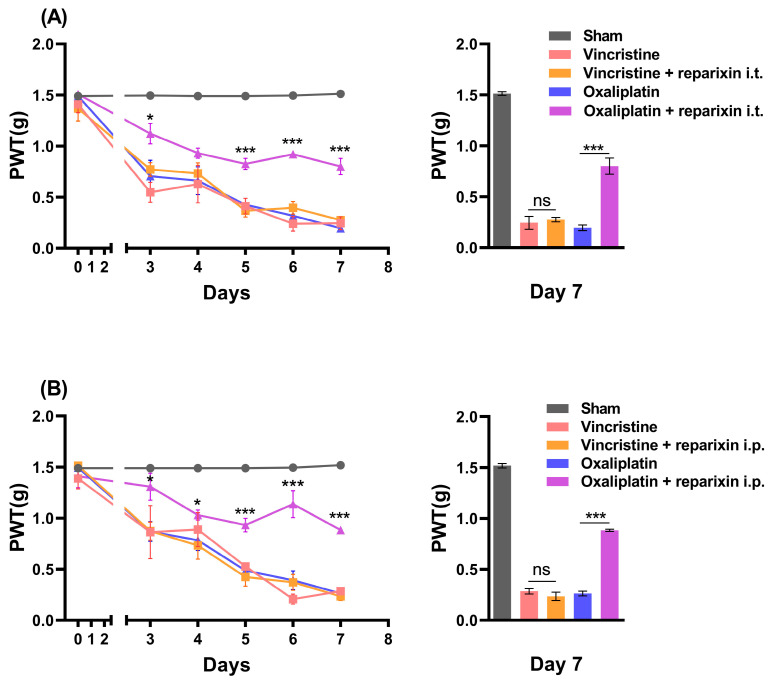
(**A**) PWT of vincristine- and oxaliplatin-treated mice with reparixin administration, measured by mechanical allodynia testing using von Frey filament. Reparixin was intrathecally injected three times on the 2nd, 4th, and 6th days (n = 5). PWT of vincristine- and oxaliplatin-treated mice with intrathecal administration of reparixin on the 7th day of the experiment. (**B**) PWT of vincristine- and oxaliplatin-treated mice with intraperitoneal administration of reparixin, measured by mechanical allodynia testing using von Frey filament (n = 4). PWT of vincristine- and oxaliplatin-treated mice with intraperitoneal administration of reparixin on the 7th day of the experiment. Vincristine-treated mice were compared to vincristine and reparixin-treated mice, and oxaliplatin-treated mice were compared to oxaliplatin and reparixin-treated mice. Results are presented as the mean ± SEM (* *p* < 0.05, *** *p* < 0.001, ANOVA followed by Bonferroni post hoc test).

In order to determine whether CXCR2 blockade in the spinal cord is involved in the development of CIPN, reparixin, a CXCR1/2 inhibitor, was intrathecally administered along with oxaliplatin or vincristine. After measuring the baseline of the PWT on day 0, reparixin was injected (n = 5) intrathecally on the 2nd, 4th, and 6th days after measuring the PWT. The PWT measured on the 7th day was 0.244 ± 0.064 g in mice with vincristine administration and 0.276 ± 0.022 g in mice with vincristine and reparixin administration, showing no significant effect of reparixin administration on vincristine-induced neuropathy. Meanwhile, the PWT following oxaliplatin-induced mechanical allodynia was 0.196 ± 0.027 g, which was significantly inhibited by reparixin administration, with a PWT of 0.800 ± 0.080 g (Figure 3A). These results suggest that CXCR2 in the spinal cord was involved in the development of oxaliplatin-induced neuropathy but not vincristine-induced neuropathy.

Next, we studied whether the intraperitoneal administration of reparixin, a drug known not to cross the blood–brain barrier (BBB) [34], could prevent CIPN (n = 4). Similar to the results of the intrathecal administration of reparixin, oxaliplatin-induced neuropathy was blocked by reparixin administration from 0.263 ± 0.023 g to 0.883 ± 0.010 g, but not vincristine-induced neuropathy from 0.285 ± 0.026 g to 0.235 ± 0.041 g (Figure 3B). This result is considered to be due to the BBB damage caused by oxaliplatin [35].

### 2.4. Inhibition of JAK2 with Ruxolitinib Prevents Oxaliplatin-Induced CIPN

**Figure 4:**
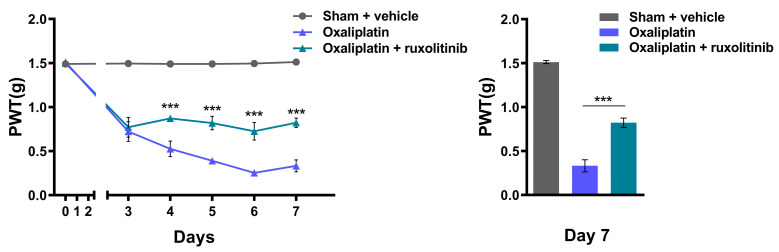
PWT of oxaliplatin-treated mice with intraperitoneal administration of ruxolitinib measured by mechanical allodynia testing using von Frey filament, and PWT measurement on the 7th day (n = 5). Oxaliplatin-treated mice compared to oxaliplatin with ruxolitinib-treated mice. Results are presented as the mean ± SEM (*** *p* < 0.001, ANOVA followed by Bonferroni post hoc test).

In order to further confirm the involvement of Janus kinase 2 (JAK2) downstream of the CXCR2 pathway [36] in the development of oxaliplatin-induced neuropathy, ruxolitinib, a JAK2 inhibitor, was administered to the oxaliplatin-treated mice (n = 5). Since ruxolitinib can cross the BBB [37], the intraperitoneal injection of ruxolitinib was accompanied by oxaliplatin administration. Ruxolitinib administration inhibited the development of oxaliplatin-induced neuropathy, and the PWT on the 7th day increased from 0.332 ± 0.068 g to 0.822 ± 0.053 g (Figure 4). This shows that the involvement of the CXCR2 pathway is associated with the development of oxaliplatin-induced neuropathy.

## 3. Discussion

In this study, we demonstrated that the development of oxaliplatin-induced CIPN, not vincristine-induced CIPN, is determined by the intraspinal CXCR2 pathway and is prevented by the blockade of CXCR2. This suggests that if CIPN, which is predicted to be caused by oxaliplatin, can be blocked in advance, treatment for cancer can be successfully performed without giving up halfway due to pain.

### 3.1. The Peripheral Neuropathy and Epidermal Change

In this study, we confirmed that vincristine and oxaliplatin not only induced peripheral neuropathy but also showed different actions in skin tissue. It is known that oxaliplatin acts with a cytotoxic effect on tumor cells by binding platinum to neuronal DNA [9] and that vincristine leads to damage in cell division and apoptosis by inhibiting the formation of mitotic spindles and the assembly of microtubules [5]. Our results show that the consecutive administration of each drug reduced the PWT, and the maximum pain response was seen 7 days after drug administration.

As regards the change in peripheral tissues, oxaliplatin is known to induce intraepidermal nerve fiber loss but no significant change in the epidermis length [38]. This finding is in accordance with our findings that both oxaliplatin and vincristine cause pain, but only vincristine increases the epidermal thickness and cell infiltration in the epidermis and dermis layer (Figure 1). In the H&E staining of the skin tissue, all the infiltrated cells in the epidermis and dermis are not inflammatory cells, but they can be qualitatively evaluated as inflammatory cells and their increase means that inflammation has occurred [39]. Kingery (2010) reported a relationship between epidermis thickening and an immune response with pain [40], implying that the mechanical allodynia developed by vincristine may partially be due to peripheral immune responses.

### 3.2. The Changes in CXCR2 mRNA Expression in the Spinal Cord

Chemokines and cytokines are involved in neuropathic pain, including CIPN [6]. Thus, we examined the mRNA expression of both lumbar DRG and lumbar spinal cord tissues, which may be directly related to the development of mechanical allodynia. CXCR2 in the spinal cord, which plays an important role in the development of oxaliplatin-induced neuropathy, and its related ligands, CXCL1, 3, and 5, were analyzed together. On the 1st day of vincristine and oxaliplatin treatment, slight changes were observed in the mRNA expression of all factors related to CXCR2 in DRG. Meanwhile, on the 7th day, CXCL1 and CXCL5 expression increased in the lumbar spinal cord, and CXCL3 and CXCL5 expression decreased in DRG after vincristine and oxaliplatin administration. These results are unlikely to be involved in the early stages (days 1~3) of CIPN development. However, these chemokine ligands in the spinal cord may be involved in persistent pain, as reported by Zhou et al. (2018) [24] and Xu et al. (2016) [41]. In the spinal cord, CXCR2 mRNA expression robustly increased on the 1st day when oxaliplatin was administered, which lasted until the 7th day, while CXCR2 expression only increased on the 7th day in the vincristine group. In neuropathic pain, CXCL1–CXCR2 interactions in the spinal cord induce mechanical allodynia [42]. Previous studies are consistent with our results. Brandolini et al. (2019) [27] and Zhou et al. (2018) [24] reported that CIPN was associated with the NF-kappa B-dependent CXCL1(IL-8)/CXCR2 signaling pathway. As CXCL3 expression by oxaliplatin administration and CXCL5 expression by both vincristine and oxaliplatin administration decreased in the spinal cord, these chemokine ligands may not be involved in the activation of CXCR2.

In addition, in our unpublished results, the spinal cord histology (H&E staining) revealed an increase in the number of cells that infiltrated the lamina I and lamina II layers only in the oxaliplatin-treated mice, which is in accordance with the infiltrated inflammatory cells in the spinal dorsal horn observed after oxaliplatin administration by Dong et al. (2022) [43] and the lack of mast cell infiltration observed after vincristine administration by Starobova et al. (2021) [44], suggesting oxaliplatin-induced inflammatory cell infiltration in the spinal cord. Although the types of cells infiltrating the spinal cord, such as mast cells, macrophages, monocytes, and microglia, have not been identified, it is thought that the infiltration of these inflammatory cells is related to the increase in CXCR2 in the spinal cord of oxaliplatin-treated mice, which may lead to mechanical allodynia. In terms of the different effects of vincristine and oxaliplatin, vincristine reduces damage to the blood–brain barrier (BBB) [45], while oxaliplatin induces the loosening of the BBB [35]. This suggests one plausible explanation for the increase in spinal CXCR2 expression in the oxaliplatin-treated mice. Collectively, in terms of CIPN development and not persistent pain, our results implied that increased CXCR2 in the spinal cord in the oxaliplatin group played a key role in the development of CIPN by oxaliplatin. However, the increased CXCR2 expression in the vincristine group on the 7th day is not considered to contribute to the development of CIPN since increased CXCR2 expression emerged after the mechanical allodynia had already been established.

### 3.3. The Role of Spinal CXCR2 in the Development of Oxaliplatin-Induced Neuropathy

Whether or not spinal CXCR2 signaling is involved in the development of CIPN, and if so, whether vincristine- or oxaliplatin-induced neuropathy is related to CXCR2, are the questions that remain. Reparixin, an inhibitor of CXCR1/2, was observed to have a preventive effect in the development of CIPN in the oxaliplatin-treated mice but not in the vincristine-treated mice when given intrathecally (Figure 3). Additionally, JAK2, which is present downstream of CXCR2 activation [46], showed a similar result when it was inhibited by the JAK2 inhibitor ruxolitinib, as shown in Figure 4. The results imply that the mechanical allodynia that developed following oxaliplatin treatment was due to the overexpressed CXCR2 in the lumbar spinal cord. The preventive effect targeting the CXCR2/JAK2 signal pathway highlights the role of spinal CXCR2 in the development of CIPN by oxaliplatin. The question remains as to why the inhibition of CXCR2 by reparixin did not have any effect on vincristine when spinal CXCR2 expression increased on the 7th day. Although the indirect inhibition of CXCR2 alleviates persistent CIPN by vincristine [24], our result indicates that the CXCR2 pathway may not be involved in the development of vincristine-induced CIPN.

The importance in our study of systemic reparixin administration is that the drug can be administered intraperitoneally rather than intrathecally, which could be a crucial difference when it comes to clinical issues, as repeated intrathecal injections can be a great burden to the patient compared to an intraperitoneal injection, a type of systemic administration. When reparixin, which cannot cross the BBB, was administered intraperitoneally, we observed a similar effect to intrathecal injection (Figure 3). Additionally, the preventive effect lasted up to the 7th day. This is believed to be in accordance with the loosening of the BBB induced by oxaliplatin administration.

### 3.4. The Clinical Implication for the Prevention of Oxaliplatin-Induced CIPN

The significant problems of CIPN derive from the fact that severe CIPN often leads to chemotherapy discontinuation. Thus, it is important to determine how the problem should be addressed based on whether the aim is to prevent the predicted pain or to alleviate the symptoms of CIPN, which lasts even after chemotherapy is finished. This study focused on the difference between the development of CIPN by vincristine and oxaliplatin administration. The significance of this study is that it differentiates the previously ambiguous chemokine pathway in CIPN development and focuses on the prevention of CIPN instead of treatment. Our findings suggest that identical chemokine pathways may have a preventive effect depending on what chemotherapeutic agent is used. Overall, even though both vincristine and oxaliplatin can lead to the same phenotypic side effects termed CIPN, and the CXCR2 pathway plays a role in CIPN, the mechanism behind the development of CIPN by vincristine and oxaliplatin seems distinct. A blockade of the CXCR2 pathway also has a unique effect, i.e., a treatment effect with vincristine-induced CIPN and a preventive effect with oxaliplatin-induced CIPN. Therefore, the appropriate countermeasures for CIPN between prevention and treatment should be determined for the different types of chemotherapeutic agents, even if a drug, such as reparixin, is used to inhibit identical CXCR2 pathways to achieve successful anticancer treatment.

## 4. Materials and Methods

### 4.1. Animals

All experimental methods were approved by the Experimental Animal Ethics Committee of Hanyang University. C57BL/6 wild-type mice (OrientBio, Sungnam, Republic of Korea), weighing 18–22 g, were used in this study. Experimental animals were housed at 22 °C and 60% humidity with 12 h light–dark cycles and fed food and water ad libitum. All animals had one week of adaptation period to the new environment before the experiment. Every animal model consisted of 4 to 5 mice, and injections were administered on the same day of and after the experiment was complete.

### 4.2. Reagents

Solutions of vincristine sulfate (Sigma Aldrich, St. Louis, MO, USA), oxaliplatin (Sigma Aldrich, St. Louis, MO, USA), reparixin (Sigma Aldrich, St. Louis, MO, USA), and ruxolitinib (Adooq Bioscience, Irvine, CA, USA) were prepared in 5% DMSO (Sigma Aldrich, St. Louis, MO, USA), 40% PEG300 (Sigma Aldrich, St. Louis, MO, USA), 5% tween 80 (Daejung Chemicals and Metals, Siheung-si, Republic of Korea), and 50% saline (Daihan Pharm, Seoul, Republic of Korea).

### 4.3. Drug Treatment

Vincristine was dissolved to a concentration of 0.1 mg/kg, oxaliplatin to 3 mg/kg, reparixin to 20 mg/kg, and ruxolitinib to 10 mg/kg for intraperitoneal administrations, and reparixin to 50 μg/kg for intrathecal administration. Intraperitoneal administration was performed for 7 consecutive days, and intrathecal administration was performed 3 times on alternative days starting from 2nd day.

### 4.4. Von Frey Test

All experiments were performed in a quiet environment by the same investigator. As a measurement of hypersensitivity to mechanical allodynia, the mice were habituated for 30 min on an iron-wired mesh (1 mm × 1 mm) and placed individually in chambers. All mice were habituated for one to two hours in the experiment room prior to testing. Mechanical allodynia was measured by applying von Frey filaments of different forces to the center of the paw while avoiding the walking pads. The response was recorded on the observation of hind paw withdrawal and or twitching with the appropriate von Frey filament scale based on the up-down method [47] and evaluated by the quantitative assessment developed by Chaplan et al. 1994 [48].

### 4.5. Real-Time PCR

Total RNA was isolated from mouse DRG and spinal cord tissue using Trizol reagent (Takara, Kusatsu, Japan). Reverse transcription was performed using 1 μg of total RNA along with random hexamer (Thermo Fisher Scientific, Waltham, MA, USA), then placed under Thermal cycler (T100 thermal cycler, Bio-Rad, Hercules, CA, USA) for 5 min at 70 °C. Master mix was prepared using Improm II 5X buffer (Promega, Madison, WI, USA), dNTP (Takara, Kusatsu, Japan), RNAsin Ribonuclease inhibitor (Promega, Madison, WI, USA), and Reverse transcriptase (Promega, Madison, WI, USA) with a cycling protocol of 25 °C for 5 min, 42 °C for 60 min, 70 °C for 15 min, and rest at 4 °C. The cDNA product was used as a template for the quantitative measurement of the amplified target gene transcripts using real-time PCR. Quantitative real-time PCR was performed with Bio-rad CFX Connect Real-Time system (Bio-Rad, Hercules, CA, USA) and Sensifast no-ROX SYBR green (Bioline, Cincinnati, OH, USA), as instructed by the manufacturer. PCR primers were ordered through Macrogen, Seoul, Republic of Korea, and sequences are as follows:

GAPDH forward, 5′-AGGTCGGTGTGAACGGATTTG-3′;
GAPDH reverse, 5′-TGTAGACCATGTAGTTGAGGTCA-3′;
CXCR2 forward, 5′-TGAGGGTCGTACTGCGTATC-3′;
CXCR2 reverse, 5′-AGTGTGAACCCGTAGCAGAA-3′;
CXCL1 forward, 5′-TCCAGAGCTTGAAGGTGTTGCC-3′;
CXCL1 reverse, 5′-AACCAAGGGAGCTTCAGGGTCA-3′;
CXCL3 forward, 5′-TGAGACCATCCAGAGCTTGACG-3′;
CXCL3 reverse, 5′-CCTTGGGGGTTGAGGCAAACTT-3′;
CXCL5 forward, 5′-CCGCTGGCATTTCTGTTGCTGT-3′;
CXCL5 reverse, 5′-CAGGGATCACCTCCAAATTAGCG-3′.

The PCR amplifications were performed as follows: denature at 95 °C for 10 s, annealing and extension at 59 °C for 45 s, repeated 40 times, followed by melting curve detection scheme.

### 4.6. Staining

Mice were anesthetized, then perfused with saline, and then with 4% paraformaldehyde. Hind paw skin tissue was obtained and placed in 30% sucrose in phosphate-buffered saline (PBS) solution for 48 h. Frozen transverse sections (12 μm-thick) were mounted on the slide and stained with hematoxylin and eosin (H&E) for histological analysis using microscope (Leica DM5000, Leica Microsystems, Wetzlar, Germany) with 20× (Leica 506503, Leica Microsystems, Wetzlar, Germany) lenses along with LAS X program (LAS version 4.0.0, Leica Microsystems, Wetzlar, Germany). Thickness of hind paw skin was measured using image J software (Image J 1.53t, Wayne Rasband and contributors National Institutes of Health, USA). The epidermis area was measured stratum basale to stratum granulosum, and the infiltration of the cells was counted in three randomly picked areas (0.01 mm^2^) per sample.

### 4.7. Statistical Analysis

All data analysis was performed using GraphPad Prism 8.0.1 software (GraphPad Prism 8.0.1, Boston, MA, USA). Differences with a *p* value < 0.05 were considered statistically significant. One-way ANOVA was used to analyze the data with Bonferroni post hoc test. Data are expressed as mean ± SEM.

## Data Availability

Not applicable.
